# Use of taste-masking product, FLAVORx, to assist Thai children to ingest generic antiretrovirals

**DOI:** 10.1186/1742-6405-3-30

**Published:** 2006-12-29

**Authors:** Torsak Bunupuradah, Siripan Wannachai, Arpa Chuamchaitrakool, Jintana Intasan, Thantip Nuchapong, Woodie Neiss, Kenny Kramm, Chitsanu Pancharoen, David Burger, Jintanat Ananworanich

**Affiliations:** 1 The HIV Netherlands Australia Thailand Research Collaboration (HIV-NAT), Bangkok, Thailand; 2 FLAVORx company, Bethesda, USA; 3 Chulalongkorn University, Bangkok, Thailand; 4 UMC St Radboud, Nijmegen, The Netherlands

## Abstract

We evaluated whether FLAVORx helped thirty Thai children take opened capsule, crushed tablets and liquid generic ARVs with more ease. All children had excellent adherence, evaluated by PACTG Standard International Questionnaire and interviewing, before and after one month of FLAVORx. Eighty percent took ARV with more ease and wish to continue FLAVORx. Strawberry was the most popular flavor.

## Background

Most children with HIV infection live in resource-limited settings. With the recent availability of generic antiretrovirals (ARV), more children are being treated. The Thai Government Pharmaceutical Organization (GPO) began producing generic ARVs in 2001. Out of about fifty thousand children living with HIV in Thailand, five thousand children are now on ARVs [1]. However, the formulations of ARVs produced by the GPO are mainly solid dosage forms for adults with only a few liquid dosage forms suitable for children. In addition, the Access to Care Program by the Thai Government provides mainly pill formulation. Children under seven years of age are usually unable to swallow pills, capsules or tablets. The HIV Netherlands Australia Thailand Research Collaboration (HIV-NAT), Thai Red Cross AIDS Research Centre, has over one hundred fifty children on ARVs, many of these children have to take opened capsule, crushed tablets or large amounts of liquid ARVs. Inappropriate formulations and bad taste have been reported to lead to poor adherence in children [2] which can in turn cause treatment failure [3-6]. We think that it is important to explore ways to make it easier for our children to take ARVs since these are long term chronic medications that children with HIV must take to survive; anything that can be done to make their circumstances easier, live happier is a virtue.

FLAVORx flavors are FDA, TGA (Australian Regulatory Agency – Therapeutic Goods Administration), HAS (Health Services Australia) and Health Canada approved. When FLAVORx was added in the proper formulations they mask the bad, bitter or acrid tastes of medications. FLAVORx has been used in the United States, Canada, Australia and New Zealand to make medicine including ARVs more palatable. FLAVORx is a non allergenic, sugar-free inert product [see more information in additional file 1, 2] that does not interfere with drug levels. Over forty million prescriptions were filled so far with FLAVORx, without any interactions reported. (Personnel communication: Kenny Kramm, President, CEO of FLAVORx company). Impact of FLAVORx on pediatric inpatients admitted to a Children's hospital has been evaluated as reported earlier [7,8]. In this study, we evaluated whether FLAVORx, a taste masking blend, helped thirty Thai children take opened capsule, crushed tablet and liquid generic ARVs with more ease.

## Patients and methods

From September to November 2005, caregivers of children with HIV infection whom were part of our prospective cohort study and taking opened capsule, crushed tablets or liquid ARVs were asked if they were interested in having their children use a taste-masking product. The first thirty children whom caregivers agreed were recruited. All caregivers understood the study and signed the informed consent form. The study protocol, HIV-NAT 015, opened label, one arm, cohort study, was approved by the Chulalongkorn Institutional Review Board.

Before the start of the study, ten flavors (strawberry, orange, banana, grapes, bubble gum, water melon, lemon, cherry, vanilla, chocolate) were mixed with the following generic ARVs, products of Thai GPO, by research staffs at The FLAVORx Research Institute and taste to find the best flavors for taste masking: Stavudine or d4T (Stavir^®^, GPO) opened capsule, Zidovudine or AZT (Antivir^®^, GPO) opened capsule and liquid, Didanosine or ddI (Divir^®^, GPO) powder, Nevirapine or NVP (Neravir^®^, GPO) crushed tablet, Lamivudine or 3TC (Lamivir^®^, GPO) crushed tablet and liquid, or Efavirenz or EFV (Stocrin^®^, Merck) opened capsule and Indinavir or IDV (Crixivan^®^, Merck) opened capsule supported by the National Access to Antiretroviral Program for People living with HIV/AIDS (NAPHA), were also tested. There were ten available flavors. The best flavors were then mixed with each ARV, tasted and scored by at least two Thai physicians and six nurses to determine the most likely popular flavors to Asians' palate. The best three flavors for each ARV were then offered to the children in the study before the other flavors were offered in order to limit the number of flavors children have to taste.

Children were followed twice, one month apart. At each visit, the caregivers were asked to complete the approved Thai version of the NIAID Pediatric AIDS Clinical Trial Group (PACTG) Standard International Questionnaire [9]. The caregivers were also interviewed using open ended questions about the child's adherence and difficulty in taking ARV, the caregiver's technique of giving ARV to the child and their attitude towards using a taste-masking product. At the first visit, children were encouraged to taste the different flavors and choose up to three flavors themselves. They can use different selected flavors with each of their medications. The study nurse instructed the caregiver on mixing procedure which were different for each child depending on the formulation and dosing of ARV. For example, one to three drops of FLAVORx plus four drops of a sweetening agent were used to mix one crushed tablet or one opened capsule in five ml of water. For the first ten children whom caregivers agreed, trough levels of NNRTI or PI were performed at both visits. Samples were drawn just prior to the next dose for trough levels of NNRTI or PI at both visits. After using FLAVORx for three days, the study nurse called the caregivers to confirm the method of mixing FLAVORx.

### Statistic analysis

Statistical analysis was performed with SPSS version 12.0 (SPSS, Chicago).

## Results

Thirty children were included. The characteristics are shown in Table 1. Mean age was 5.2 ± SD 1.9 years with forty percent boys. Most had mild or moderate HIV symptoms, CDC category [10] A: B: C was 10:12:8. The median CD4 was 25% (IQR 17–30). Mean weight was 16.2 ± 4.1 kilograms. Mean height was 102.8 ± 10.9 centimeters. All children were using generic NRTIs; most combining with generic NVP, branded EFV or Kaletra. The caretakers were fourteen mothers, five fathers, six grandmothers and five aunts.

**Table 1:** Demographic Data and Results

**No.**	**gender**	**Age (y)**	**Wt (Kg)**	**Ht (cm)**	**CDC**	**CD4% (cell/mm^3^)**	Regimen*	**Chosen flavors**		NNRTI Trough level **(mg/L)		**Reason of discontinue FLAVORx**
									
								**VisitI**	**VisitII**	**VisitI**	**VisitII**	
1	F	6.7	16	112.5	B	13%(277)	**AZT **1cap+7 mlX2 **3TC **1/2 tabX2 **NVP **3/4 tabX2	S	x	3.94	4.19	Spicy taste
2	M	5.5	17	111	B	39%(1108)	AZT 1 cap +6 mlX2 3TC 7 mlX2 **NVP **3/4 tabX2	S, O	x	14.80	7.01	Child prefer to mix ARV with water
3	F	3.5	12.5	92.5	A	7%(342)	3TC 5 mlX2 **EFV **200 mg **d4T **1/3 tabx2	O	x	11.88	8.29	Spicy taste
4	F	4.2	14	97	A	10%(210)	**AZT **1 cap +5 mlx2 **3TC **6 mlx2 **EFV **200 mg	S	S	2.14	1.55	
5	M	4.8	17.2	106	A	24%(1122)	**AZT**1 cap +7 mlX2 **3TC **1 tab X2 **NVP **3/4 tabX2	S, O	S,G	5.66	5.60	
6	M	5.3	12.5	93	B	6%(459)	**AZT **1 cap +4 mlX2 **3TC **5 mlx2 **NVP **1/2 tabx2	S	S	3.05	3.45	
7	F	4.5	14	99	C	17%(356)	**AZT **1 cap +5 mlX2 ddI(170 mg) 1 × 1	S,O,G	G	-	-	
8	M	4	15	96	A	42%(2278)	**AZT **1 cap +5 ml **3TC **6 mlx2 **EFV **200 mg	S	x	1.93	2.45	Vomiting
9	F	7.5	29.6	126	A	29%(614)	AZT 2 cap X2 3TC 1 tab X2 **EFV **350 mg	S	x	-	-	Child prefer to mix ARV with water
10	M	4.1	15	99	C	22%(945)	**AZT**1 cap +5 mlX2 **3TC **7 mlX2 **EFV **250 mg	S,O	S	3.18	0.00 Repeat ***	
11	F	4.6	23	106	A	25%(1224)	3TC 1 tab x2 EFV 300 mg **ddI**(115) 2 × 1	S	S	1.40	3.78	
12	M	1	11.5	84.5	C	38%(1732)	**3TC **4 mlx2 **NVP **1/2 tab X2 **d4T **10 mgx2	O	S,G,B	3.51	4.30	
13	F	4.8	14.1	97	A	35%(1428)	**AZT **1 cap +5 mlX2 **3TC **1 tabX2 **EFV **250 mg	S	S,B			
14	F	2	10.2	83	B	24%(761)	AZT 1 cap +2 ml × 2 3TC 5 ml × 2 **Kaletra **1.4 ml × 2	O	S			
15	F	4.7	11.6	93	B	15%(250)	**3TC **6 mlX2 **NVP **1/2 tabX2 **d4T **15 mgx2	S	S			
16	M	4	13.9	95.5	C	24%(960)	**AZT **1 cap +4 mlx2 **3TC **6 mlx2 **EFV **200 mg	S	S			
17	F	5.5	14	104	B	22%(591)	**AZT **1 cap +4 mlX2 **3TC **1/2 tab x2 **NVP **3/4 tabx2	S	S			
18	M	4.3	15.1	103	C	26%(1261)	**AZT **1 cap +5 mlX2 3TC 7 mlX2 EFV 200 mg	S	S			
19	M	7.7	17.5	105	B	23%(915)	3TC 1/2 tabX2 **EFV **250 mg d4T 15 mgX2	O,G	G			
20	F	5.4	20	105.5	B	41%(1137)	**AZT **1 cap +5 mlX2 **3TC **1tabX2 **EFV **250 mg	S,O	B,A			
21	F	9.8	18	119	B	27%(1086)	**AZT **1 cap +8 mlX2 3TC 1 tabX2 EFV 300 mg	O,G	G			
22	F	8.8	18	116.5	B	6%(112)	**AZT **1 cap +8 mlX2 3TC 1/2 tabX2 NVP 3/4 tabX2	S	G			
23	F	1.8	10	82	A	33%(1933)	AZT 1 cap +1 mlx2 3TC 5 mlX2 **Kaletra **1.4 ml × 2	S,O	O			
24	M	8	22.6	119	B	25%(809)	3TC 1 tabX2 d4T 1/2 tabx2 **NVP **3/4 tab x2	S,O	x			Child prefer to mix ARV with water
25	F	4	15.2	94	B	28%(817)	3TC 1/2 tabX2 **EFV **200 mg d4T 15 mg	S,B	S,B			
26	F	6.3	16.9	109	C	26%(723)	**AZT **1 cap +5 mlX2 3TC 1/2 tabX2 NVP 3/4 tabX2	O	O			
27	F	6.7	18.3	110	C	40%(1109)	3TC 7 mlX2 **EFV **250 mg d4T 15 mg	S,O	B			
28	M	4.5	15	102.5	A	28%(1076)	3TC 1/2 tabX2 EFV 200 mg **d4T **15 mgX2	G	G			
29	F	6	19.2	110	A	12%(551)	**AZT **1 cap +8 mlX2 3TC 1/2 tab X2 NVP 3/4 tabX2	S,O	G			
30	M	5.7	20	114	C	17%(345)	**AZT **1 cap +8 mlX2 3TC 1 tabX2 EFV 300 mg	S	S			

* The **bolded **drugs were mixed with FLAVORx

** compare to NNRTI target serum level: for NVP is 3.4 mg/l, EFV is 1.0 mg/l (Ref: John G. Bartlett, John Hopkins University School of Medicine, Pocket Guide to adult HIV/AIDS treatment, Jan 2005.

*** Repeat trough level without using FLAVORx = 3.74 mg/l

Chosen Taste: S = strawberry, O = orange, G = grape, B = banana, A = apple, X = children or parent did not want to continue FLAVORx

Cap = capsule, tab = tablet

At the first visit, all thirty caregivers answered that the child had never missed any ARV dose and that they did not experience problems with giving the child the ARV on time everyday. However, during the interview, most caregivers reported that the child disliked taking ARVs because of the bitter taste especially generic AZT syrup, 3TC syrup, ddI powder, NVP crushed tablet and EFV opened capsule. The techniques caregivers used varied from using alarm clock, having the child drink fruit juice immediately after the dose, mixing ARV with syrup, honey or food, forcing the child to take ARV and bribe the child with treats. The flavors chosen at the first visit were strawberry, orange and grape in twenty three, fifteen and four children respectively.

At the final visit, caregivers gave the same answers for the PACTG adherence questionnaire with no reported problems or occurrence of poor adherence. From the interview, twenty four caregivers reported that, after using FLAVORx, their children had an easier time taking ARV FLAVORx. All of them reported that their children liked the nice flavor of FLAVORx and seven of twenty four children said the medications tasted sweeter. Twenty-four of thirty (eighty percent) children said that they liked FLAVORx and wanted to continue using it. Fifteen children (fifty percent) chose to use the same flavor; strawberry (thirteen), orange (two), grapes (four), banana (one), and nine wanted to try new flavors; strawberry (six), orange (ten). Six children who didn't want to continue using FLAVORx; caregivers of three children found it too difficult, two children (on orange and strawberry flavors) reported burning sensation on the tongue, and one child had repeated vomiting immediately after taking FLAVORx (strawberry flavor).

Therapeutic drug monitoring in the first ten children showed trough levels above the target serum levels [11] at the first and final visits in all except one boy who had undetectable level at the final visit. This boy was taking EFV with strawberry and orange flavors. The mother reported excellent adherence and more ease of taking ARV after using FLAVORx. Repeat trough level in this child was 3.74 mg/l; however, he was not taking FLAVORx at that time.

## Discussion

In eighty percent of children participants, FLAVORx helped them take ARVs with more ease by masking the bitter taste of ARV. Strawberry, orange and Grape flavors were the most popular. FLAVORx did not affect adherence as full adherence was reported in all children despite the problem of bitter ARV. In other parts of the world, children may not be as willing to take bitter ARV. FLAVORx did not affect ARV trough levels in nine of ten children.

The using of taste masking products has been shown to help children take medications. Robert Wood Johnson Children's Hospital in New Jersey found improvement in hospitalized children's willingness to take medications [7,8]. In the United States, the most popular flavor is bubble gum. This was not selected by any of our patients. Palatable tastes are probably affected by the typical food flavors in each country. This is the first study of FLAVORx in Asian children. It is likely that children from other Asian countries would prefer the same flavors as our children.

After using FLAVORx, most caregivers reported that their children were happier during medication taking time which fulfilled our goal for performing this study. A few caregivers found the mixing procedure too difficult. Therefore, this product may not be ideal to use in children of caregivers who are unable to follow mixing procedures. Although we cannot be certain, we suspect that the child with undetectable efavirenz level at the final visit did not take his medication. As an inert product, FLAVORx should not effect on drug levels and it did not in nine of ten children. Any interaction would have also unlikely to cause an undetectable level.

There were two children (on orange and strawberry flavors) felt a burning sensation on the tongue, which may be due to excess use of flavoring agent or used wrong combination of flavors for that child's sense of taste. There was one child had repeated vomiting immediately after taking FLAVORx (strawberry flavor) which could be due to the child's sense of taste did not agree with this flavor choice or the flavoring needed to be adjusted or the child had a concomitant illness. After the study, we found that at times some children went back to using the marketed highly concentrated sugar syrup called Hale's Blueboy to sweeten the medications. This is much sweeter than FLAVORx but the high sugar content is not good for their dental health.

In summary, masking the taste of ARV by using FLAVORx helped Thai children take ARV with more ease. This may be explored in other countries especially where adult ARV formulations are used in children.

## Supplementary Material

Additional file 1 Product specific sheet code number 710-381. FLAVORx Strawberry Cream Flavor. Flavoring nomenclature: N&A Strawberry Cream Flavor. Non-Flavoring Ingredients: Propylene Glycol, Ethyl Alcohol, Water, Triacetin. No allergen, pH: N/A, colorless to pale red, water solubility, no alcohol range by volume, recommended storage 7.2- 26.7°C, absent coliform count. Click here for file

Additional file 2 Product specific sheet code number 711-218. FLAVORx Orange Creamsicle Flavor. Flavoring nomenclature: N&A Orange Creamsicle Type Flavor. Non-Flavoring Ingredients: Propylene Glycol, Ethyl Alcohol. No allergen, pH: N/A, yellow-green color, water solubility, no alcohol range by volume, recommended storage 7.2- 26.7°C, absent coliform count. Click here for file
