# Supplementary material for: Use of taste-masking product, FLAVORx, to assist Thai children to ingest generic antiretrovirals
Source: AIDS Res Ther. 2006 Dec 29;3:30. doi: 10.1186/1742-6405-3-30 (PMC1770927; doi:10.1186/1742-6405-3-30)
Supplement: Additional file 1 — Product specific sheet code number 710-381. FLAVORx Strawberry Cream Flavor. Flavoring nomenclature: N&A Strawberry Cream Flavor. Non-Flavoring Ingredients: Propylene Glycol, Ethyl Alcohol, Water, Triacetin. No allergen, pH: N/A, colorless to pale red, water solubility, no alcohol range by volume, recommended storage 7.2- 26.7°C, absent coliform count. [file 1742-6405-3-30-S1.pdf]

Flavormatic Industries, Inc.
230 All Angels Hill Road
Wappingers Falls, New York 12590-3329
Tel: +1 (845) 297.9100 <> Fax: +1 (845) 297.2881
e-mail: qaqclab@flavormatic.com

Product Specification Sheet

SOP Category No: 21 **SOP No:** 21:710-381
Created By: Richard Febles **Revision No/Date:** 1 28-Apr-2005
Reason for Revision: N/A

This product is a compounded mixture, and the specific chemical identities of the ingredients are considered by FLAVORMATIC INDUSTRIES, INC. to be a Trade Secret and are withheld in accordance with the provisions of 1910.1200 of Title 29 of the Code of Federal Regulations and all other pertinent International Laws and Treaties. All flavor ingredients contained in this flavor are approved for use in a regulation of the U.S. Food & Drug Administration (FDA) and/or are listed as being Generally Recognized as Safe (FEMA-GRAS) on a reliable industry association list.

Flavormatic Code Number: **710-381**

Flavormatic Nomenclature: **N&A Strawberry Cream Flavor**

Non-Flavoring Ingredients: Propylene Glycol,Ethyl Alcohol,Water, Triacetin
Label Statement: N&A Flavor
Does this Contain Allergens: No

Specific Gravity (25/25°C): 0.9939 **Range:** 0.9790 to 1.0088
Refractive Index (25°C): 1.429 **Range:** 1.4076 to 1.4504
Flash Point (TCC): 75 - 85°F (23.9 - 29.4°C)
Brix: N/A
pH: N/A
Color: Colorless to pale red
Taste/Aroma: As per Standard
Solubility: Water
BATF/TTB No: N/S
Alcohol Range by Volume: N/S

Recommended Storage(1): 45.0° - 80.0°F (7.2° - 26.7°C)
Shelf Life(2): 9 Months

Total Aerobic Plate Count: <5000/g (CFU/g)
Total Coliform Count: Absent (CFU/g)
Total Yeast & Mold Count: <50/g (CFU/g)

(1) Store in tightly sealed containers under dry, cool conditions. Store away from any sources of light and heat. Product may oxidize over time - it is important to keep the container tig

(2) Product may oxidize over time - it is important to keep the container tightly sealed when not in use. In regards to shelf life. If the product is opened-closed frequently, it will not last past the stated shelf life and in most instances, due to oxidation, it will last considerably shorter. We strongly recommend re-evaluation of the product throughout its specified shelf life. If flavors are opened and closed frequently, we strongly recommend purging the container with Nitrogen to minimize the possibility of the flavor oxidizing. For customers with different storage conditions, we strongly recommend validating your shelf life based upon your storage conditions.

Flavormatic makes no express warranties and disclaims any implied liability of merchantability or fitness for use or any other implied warranties, other than goods conform to the description thereof or on the face hereof. We recommend that all products be re-evaluated by organoleptic or analytical means upon expiration of the stated shelf life period. Continued use of the flavor beyond the specified shelf life and the determination that the flavor remains usable is the responsibility of the purchaser.

The information contained herein is based on data considered to be accurate and reliable. However, the data is provided without any warranty, expressed or implied regarding its correctness or accuracy. It is the users responsibility both to determine safe conditions for use of this product, and to assume liability for loss, injury, damage or expense resulting from the improper use of this product. Other states, provinces and municipalities may have specific regulations concerning this product which are not reflected in this document. The user should review these regulations to ensure full compliance. If you have any questions or comments, please address them to Flavormatic C/O above address

FLAVORMATIC Fax:1 Apr 28 '05 10:23 P.07/07
